# Progress in Neurology

**Published:** 1901-11-30

**Authors:** 


### PROGRESS IN NEUROLOGY.

**Myasthenia Gravis.**—Up to the present time no pathological changes have been found, either in the nervous system or muscles, which might account for the condition. Laquer and Weigert [1] have, however, recently published an account of, clinically, a typical case of myasthenia gravis, in which definite changes were found on autopsy. In the situation of the thymus gland a tumour was found, which, microscopically, was found to consist of small cells, lymphoid cells, larger epithelioid cells, and Hassell's bodies. In the deltoid and diaphragmatic muscles, it was found that at many points both in the sheath of the muscles and between the muscle-fibres, there was a great invasion of small lymphoid cells which closely resembled those found in the thymus tumour, and to a lesser extent the larger epithelioid cells were also present. The muscle fibres themselves, however, appeared to be normal. The combination of myasthenia with disease of the thymus is noteworthy, but it is hardly probable that such an association is at all common in view of detailed accounts of the pathological condition of previous cases.

**Progressive Muscular Atrophy.**—Williamson [2] has recorded the pathological changes in a case of progressive muscular atrophy. Lateral sclerosis has so often been found post-mortem in such cases that some writers have grouped this affection and amyotrophic lateral sclerosis as one and the same disease. But Beevor, J. B. Charcot, and others have published cases in which the lateral pyramidal tracts were unaffected, and this one is yet another. The disease had lasted 20 years. There was slowly progressing muscular atrophy, with paralysis of the small muscles of the hands, the forearm, and upper arm muscles, and finally the muscles of the neck. Sensation was always unaffected, and the legs, bladder, and rectum were normal. Thus clinically there were no symptoms to indicate any affection of the lateral pyramidal tracts at the end of 20 years. Both by Marchi's and Weigert's methods the white matter of the spinal cord was found to be normal. In the middle and lower cervical region the anterior cornual cells had disappeared, and the fine delicate nerve fibres were greatly diminished in numbers, whereas the nerve fibres entering the posterior horns from the posterior roots were quite normal.

**Tumours of the Frontal Lobe of the Brain.**—Höniger [3] remarks that a diagnosis may be made when there is disturbance of the psychical functions. The mental alteration may express itself in the form of loquacity and a tendency to joking combined, and this condition of irrepressible quibbling is especially marked when the left frontal lobe is the seat of the tumour. Another trait is an ataxic gait which resembles that of a drunken person, which is attributable to a weakness or paresis of the trunk muscles in the lumbo-sacral region, and sometimes to a spasmodic contraction of these muscles. A permanent paralysis of these muscles follows as the tumour grows and enlarges, involving the cortex of the superior frontal convolution. In proportion as the tumour approaches the third left frontal convolution, speech disturbances predominate. Other symptoms develop if the tumour approaches the motor area. If the tumour starts in the basal aspect of the lobe, its proximity to the olfactory and optic nerves may give rise to disturbances of those nerves. It will be remembered that Munk by experimental ablation of the frontal lobes in dogs and monkeys produced paralysis of the lumbo-sacral muscles. A case is given where the diagnosis of frontal tumour was made on the basis of paresis of the lumbo-sacral muscles, with characteristic disturbance of gait; and the diagnosis was subsequently verified by autopsy.

**Peripheral Neuritis.**—Buzzard [4] has analysed the last 120 cases of so-called "alcoholic" peripheral neuritis admitted to the National Hospital for the Paralysed and Epileptic, to determine the factors in its causation. The recent arsenic epidemic in the North has led some to think that ethyl alcohol *per se* cannot produce neuritis, and that the real cause at work is in every case arsenic. Others, again, are of opinion that "alcoholic" neuritis only occurs in beer drinkers and not in pure spirit drinkers. Investigation of the above-mentioned records showed that, in London, alcoholic neuritis is more often found as a sequel of spirit drinking than in other forms of alcoholism, and the clinical evidence is antagonistic to the idea that arsenic is the cause of alcoholic neuritis. In London, though there is a large beer-drinking community, neuritis was very seldom found in persons who drank beer only; it was among the consumers of pure spirits, either alone or in conjunction with beer, that neuritis was most frequently found. In a third of the cases trophic disturbances were found. The skin of the hands and feet was glossy and thin, and this was accompanied by a striated or brittle condition of the nails. These skin changes are due to disturbed nutrition brought about by the changes in the lower neurons, and are quite distinct from the primary cutaneous changes in arsenical poisoning. It is deserving of mention that not a single case of herpes or erythromelalgia was found in the whole series, whereas among arsenical beer drinkers both of these phenomena are by no means uncommon. This evidence is sufficient to negative the opinion which has lately been put forward that ethyl alcohol *per se* is incapable of causing "alcoholic" peripheral neuritis.

**Paralysis of the Cervical Sympathetic.**—Stewart [5] records a case of paralysis of the cervical sympathetic, due to a wound by a Mauser bullet. The patient, a soldier, was shot whilst lying on his face firing at the enemy. The bullet entered the left side of his neck an inch and a half below the mastoid process, and came out through the seventh right interspace in the posterior axillary line. It thus passed in front of the vertebral column. The right arm was immediately paralysed, but recovered nearly completely in about a month. There was, however, an enduring paralysis of the right cervical sympathetic, marked by the following signs:—Narrowing of the right palpebral fissure, a slight sinking back of the right eyeball, and a small pupil which contracted normally on convergence to light, whilst it did not dilate when shaded. The most interesting feature was an absence of sweating on the right side of the face, scalp, neck, trunk, down to the level of the third dorsal vertebra, and the whole of the right upper limb; whilst the rest of the body surface sweated profusely. The above-mentioned area was bounded exactly by the middle line. The lesion was not one merely of the vasomotor nerves for pilocarpin—which stimulates sweat-nerves but has no action on vaso-dilutors—failed to produce sweating on the affected side.

**Hereditary Chorea.** — Lannois, Paviot, and Mouisset [6] have investigated the pathological anatomy of a case of chorea which had lasted nearly 30 years. The patient's father had died at the age of 80 years from the same complaint, and the patient's only brother, aged 65, was a sufferer from chronic chorea for many years. The patient's age at death was 68. In the ascending frontal convolution there was an infiltration of small round cells in the molecular layer, but the blood vessels were normal. These cells were in nests of two, three, or four, and in the pericellular sacs of the larger nerve cells they were numerous and conspicuous. The pyramidal cells showed an excess of yellow pigment in proportion to the age of the patient. No degenerated nerve fibres could be found. The lesion then was one of the cerebral cortex and from its nature it appeared to be incurable.

**Chorea.**—Lannois [7] has used sodium cacodylate in cases of chorea with great success. The drug was given subcutaneously in doses from $\frac{1}{6}$ to $\frac{1}{3}$ grain. The administration was usually continued for five days, and began again after an interval of four or five days. Three weeks' treatment sufficed to cure an intense chorea in a girl 15 years of age. In a severe case of chorea inseniens a fortnight's treatment caused distinct improvement of the physical and mental condition. Other cases also are recorded. In all cases the ordinary treatment had been previously tried without benefit.

[1] Neurol. Centralblatt, July. [2] Lancet, July 6. [3] Münch Med. Woch, May 7. [4] Lancet, June 8. [5] Brit. Med. Jour., June 8. [6] Revue Neurol., May 15. [7] Lyon Méd., Jan. 27.

